# Smoking in preeclamptic women is associated with higher birthweight for gestational age and lower soluble fms-like tyrosine kinase-1 levels: a nested case control study

**DOI:** 10.1186/1471-2393-11-91

**Published:** 2011-11-10

**Authors:** Susan R Kahn, Nisha D Almeida, Helen McNamara, Gideon Koren, Jacques Genest, Mourad Dahhou, Robert W Platt, Michael S Kramer

**Affiliations:** 1 Department of Medicine, McGill University, Montreal, Quebec, Canada; 2 Department of Epidemiology and Biostatistics, McGill University, Montreal, Quebec, Canada; 3 Department of Obstetrics and Gynecology, McGill University, Montreal, Quebec, Canada; 4 Department of Pediatrics, McGill University, Montreal, Quebec, Canada; 5 Motherisk Program, Hospital for Sick Children, Toronto, Ontario, Canada

## Abstract

**Background:**

Smoking paradoxically increases the risk of small-for-gestational-age (SGA) birth but protects against preeclampsia. Some studies have reported a "U-shaped" distribution of fetal growth in preeclamptic pregnancies, but reasons for this are unknown. We investigated whether cigarette smoking interacts with preeclampsia to affect fetal growth, and compared levels of soluble fms-like tyrosine kinase-1 (sFlt-1), a circulating anti-angiogenic protein, in preeclamptic smokers and non-smokers.

**Methods:**

From a multicenter cohort of 5337 pregnant women, we prospectively identified 113 women who developed preeclampsia (cases) and 443 controls. Smoking exposure was assessed by self-report and maternal hair nicotine levels. Fetal growth was assessed as z-score of birthweight for gestational age (BWGA). sFlt-1 was measured in plasma samples collected at the 24-26-week visit.

**Results:**

In linear regression, smoking and preeclampsia were each associated with lower BWGA z-scores (β = -0.29; p = 0.008, and β = -0.67; p < 0.0001), but positive interaction was observed between smoking and preeclampsia (β = +0.86; p = 0.0008) such that smoking decreased z-score by -0.29 in controls but increased it by +0.57 in preeclampsia cases. Results were robust to substituting log hair nicotine for self-reported smoking and after adjustment for confounding variables. Mean sFlt-1 levels were lower in cases with hair nicotine levels above vs. below the median (660.4 pg/ml vs. 903.5 pg/ml; p = 0.0054).

**Conclusions:**

Maternal smoking seems to protect against preeclampsia-associated fetal growth restriction and may account, at least partly, for the U-shaped pattern of fetal growth described in preeclamptic pregnancies. Smoking may exert this effect by reducing levels of the anti-angiogenic protein sFlt-1.

## Background

Preeclampsia, a hypertensive disorder that occurs in 2-7% of pregnancies, is an important cause of fetal and maternal morbidity and mortality [1]. Paradoxically, while maternal cigarette smoking increases the risk of a number of pregnancy complications, including miscarriage, preterm birth and small-for-gestational-age (SGA) birth, smoking has consistently been shown to be associated with a ~30% reduction in the risk of preeclampsia [2,3]. The mechanism for this protective effect is unclear, but may involve direct effects of smoking byproducts nicotine or carbon monoxide in inhibiting placental cytokine production, placental vascular constriction or oxidative stress [4,5], factors that have been implicated in the pathophysiology of preeclampsia [6]. Furthermore, it was recently reported that both in normal and preeclamptic pregnancies, smoking is associated with lower maternal plasma concentrations of soluble fms-like tyrosine kinase-1 (sFlt-1) [7], a circulating anti-angiogenic protein that induces endothelial dysfunction and is found in higher concentrations in pregnant women who subsequently develop preeclampsia [8].

In preeclamptic pregnancies, fetal growth has been described as having a "U-shaped" distribution, with an increased risk of both low birthweight and high birthweight babies [9]. In a case-control study nested within a large multicenter cohort of pregnant women whose primary aim was to evaluate the link between thrombophilia and preeclampsia, we performed secondary, hypothesis-generating analyses to assess whether maternal cigarette smoking, measured by self-report and by hair nicotine levels, modifies the association between preeclampsia and fetal growth. In addition, we examined the relationship between plasma sFlt-1 levels, smoking and preeclampsia.

## Methods

The Montreal Preeclampsia Study [10] is a case-control study nested within a prospectively recruited cohort of 5337 pregnant women who were participants in the Montreal Prematurity Study, a large multicenter study on causal pathways of preterm birth [11]. At the time of providing informed consent for the preterm birth study, the preeclampsia study was explained and women were asked to provide signed informed consent on a separate consent form. Before both studies were started, they received approval by the research ethics committees of the four participating hospital centers (Hôpital St. Luc; Hôpital Maisonneuve Rosemont; Royal Victoria Hospital and Jewish General Hospital [approved jointly by McGill University Faculty of Medicine institutional review board]) Our research conformed to the Helsinki Declaration (http://www.wma.net/en/30publications/10policies/b3/17c.pdf) and to local legislation.

### Recruitment of cohort and follow-up to time of delivery

#### Patients and study setting

Trained research assistants approached consecutive pregnant women at the time they presented for routine ultrasound examination (most subjects), prenatal blood drawing, or first- or second-trimester obstetric clinic visits at four large maternity hospitals affiliated with McGill University or l'Université de Montréal in Montréal, Canada. Women aged ≥18 years at the expected date of delivery who spoke and understood French or English and had a singleton fetus were eligible for enrolment in the study. Women with severe chronic illness (other than hypertension, asthma or diabetes) requiring ongoing treatment, placenta previa, history of incompetent cervix diagnosed in a previous pregnancy, impending delivery, or a fetus affected by a major anomaly were excluded. Among these excluded women, reasons for exclusion and data on maternal education were recorded whenever possible.

#### Study visit at 24-26 weeks

Women who consented to participate were asked to return for a study visit at 24-26 weeks of gestation to obtain data on sociodemographic characteristics, medical, obstetric and family history, and cigarette smoking. Subjects were asked if they had ever smoked, if they had smoked (defined as one or more cigarettes per day) during the pregnancy, and if so, the average number of cigarettes smoked per day.

After the interview, non-fasting blood samples were obtained by venipuncture and immediately placed on ice. Samples were centrifuged, and plasma was aliquoted into cryovials that were frozen at -80°C.

#### Identification of cases of preeclampsia and selection of control subjects

Cohort women were followed until delivery. During the admission for delivery, each study woman identified as having had hypertension or preeclampsia during pregnancy or after delivery underwent a detailed interview and chart review by trained study staff to assess whether diagnostic criteria for preeclampsia were met. Preeclampsia was diagnosed according to the 1997 Canadian Hypertension Society criteria and further classified as severe or early onset (Table 1**) **[12]. All cases of preeclampsia (n = 113) were independently verified by two study investigators (SK and HM).

**Table 1:** Classification of preeclampsia among cases (N = 113)

	Total	**Early-onset**^**b**^	**Severe**^**c**^
**Canadian Hypertension Society classification^a^**			
Gestational hypertension with proteinuria without adverse conditions	12 (11%)	3 (8%)	0 (0%)
Gestational hypertension with proteinuria with adverse conditions	49 (43%)	16 (46%)	19 (59%)
Gestational hypertension without proteinuria with adverse conditions	49 (43%)	14 (40%)	10 (31%)
Chronic hypertension with superimposed preeclampsia	3 (3%)	2 (6%)	3 (10%)

**TOTAL**	**113 (100%)**	**35 (100%)**	**32 (100%)**

**^a ^**Diagnostic criteria used for preeclampsia and non-preeclamptic hypertensive disorders of pregnancy were adapted from the 1997 Report of the Canadian Hypertension Society Consensus Conference [12]. Gestational hypertension was defined as diastolic hypertension (≥ 90 mm Hg) on two occasions at least 4-6 hours apart that developed after 20 wks gestation. Proteinuria was defined as protein excretion of ≥ 0.3 g/day in 24-hour urine collection or positive dipstick result ≥ 2+. Adverse conditions were defined as convulsions (eclampsia), diastolic pressure > 110 mm Hg, platelet count < 100,000 × 10^9^/L, oliguria, protein excretion ≥ 3 g/day, pulmonary edema, elevated liver enzymes, severe nausea and vomiting, frontal headache, visual disturbances, persistent abdominal pain in right upper quadrant, chest pain or shortness of breath, suspected abruptio placentae, HELLP syndrome, intrauterine growth restriction, oligohydramnios, or absent or reverse umbilical artery end diastolic flow, as detected by Doppler velocitometry. Chronic hypertension with superimposed preeclampsia was defined as known chronic hypertension associated with further worsening of blood pressure and protein excretion ≥ 3 g/day after 20 weeks gestation. Non-preeclamptic hypertensive disorders (criterion for exclusion as case or control) included gestational hypertension, defined as diastolic hypertension (≥ 90 mm Hg) that developed after 20 wks gestation but without proteinuria or adverse conditions, and chronic known hypertension, defined as diastolic hypertension (≥ 90 mm Hg) that predated pregnancy or was diagnosed before 20 wks gestation, with or without proteinuria.

^b ^Early-onset preeclampsia defined as occurrence at < 34 weeks gestation [18]

^c ^Severe preeclampsia defined as presence of any of the following: seizure, HELLP, proteinuria ≥ 3 g/24 hours or diastolic blood pressure > 110 mm Hg [18]

Primarily for recruiting convenience, controls (n = 443) were study women who delivered at the same hospital closest in calendar time to a case, but did not have preeclampsia. As controls partially overlapped with those of the concurrently running preterm birth study [11], 3-4 eligible controls were available per case of preeclampsia. Study women who had onset of hypertension during pregnancy but did not meet criteria for preeclampsia were ineligible to be cases or controls and were excluded from all analyses.

### Postpartum procedures for cases with preeclampsia and control subjects

After delivery, study staff collected data from the mother's and infant's hospital chart and by interview on infant birthweight, sex, placental weight, gestational age and pregnancy course since the 24-26 week study visit.

Infant birthweight and placental weight were recorded as the weights obtained in the delivery room. Gestational age was determined using both the subject's last menstrual period (LMP) as well an ultrasound estimate usually obtained at 16-20 weeks gestation. If the gestational age estimates using the two methods differed by more than 7 days, the ultrasound estimate was used; otherwise, the LMP estimate was chosen. During the postpartum interview, subjects were asked if they had smoked cigarettes (defined as one or more cigarettes per day) since the prenatal interview, and if so, the average number of cigarettes smoked per day.

#### Laboratory analyses

After delivery, cases and controls had a small amount of maternal hair cut from the posterior scalp, sealed in a labeled envelope and sent in batches to Dr. Koren's laboratory in Toronto to test for nicotine concentrations, using previously described techniques [13]. Results are expressed in nanograms of nicotine per milligram of hair.

Plasma samples of cases and controls were retrieved and thawed. sFlt-1 concentration was measured in Dr. Genest's laboratory using commercial ELISA kits from R&D systems (Minneapolis, MN). Results are expressed in pg/ml. The sFlt assay is specific for human sFlt-1, with sensitivity of 5 pg/ml. As reported by the manufacturer, intra-assay and inter-assay coefficients of variation are 3.2% and 5.5%, respectively.

All laboratory analyses were performed blindly without identifying the source of each specimen as a case or a control.

### Statistical analysis

Data on demographic and medical characteristics, self-reported cigarette smoking, pregnancy outcome, and hair nicotine and plasma sflt-1 concentrations were compared in cases and controls using t-tests for continuous variables and chi-square tests for categorical variables. Hair nicotine and plasma sFlt-1 were examined both as continuous and quantile (divided at the median and by quartiles, respectively) variables. A *P *value of < 0.05 was considered statistically significant.

Fetal growth was assessed on a continuous scale as the z-score of birthweight for gestational age (BWGA), using the following formula: z = (observed birthweight - mean birthweight)/SD, where mean and SD were based on published Canadian population-based standards, stratified by infant sex and gestational age in completed weeks [14]. SGA and LGA births were defined as having birthweight below the 10^th ^percentile and above the 90^th ^percentile, respectively, using the same Canadian population-based standards used to obtain the BWGA z-scores.

Multiple linear regression was used to test associations between measures of maternal tobacco smoke exposure, preeclampsia, and z-score of BWGA. Models were constructed in which the dependent variable was fetal growth represented by z-score of BWGA, and the independent variables were preeclampsia, maternal tobacco smoke exposure (self-reported smoking status, or log-transformed hair nicotine concentration), and an interaction term for preeclampsia and smoking exposure. As the distribution of hair nicotine was right skewed, this variable was log-transformed (natural log) to normalize the distribution. In order to retain 0 values, we added a constant (0.1) that best normalized the distribution after log-transformation.

Because birthweight and fetal growth are known to be influenced by placental weight, we also constructed models adjusted for placental weight, represented by the z-score of placental weight for gestational age (PWGA) using the following method, described by McNamara [15,16]: z = (observed placental weight at a given gestational age - mean placental weight for that gestational age)/SD, where mean and SD were based on singleton deliveries (excluding infants with congenital anomalies) between 1978 and 2001 in the McGill Obstetrical and Neonatal Database [17], stratified by ultrasound-confirmed gestational age.

Finally, the above models were further adjusted for age, pre-pregnancy BMI, maternal language other than French or English, diabetes preceding or during pregnancy, chronic hypertension and parity (nullipara vs. other).

All analyses were performed using SAS version 9.1 (SAS Institute, Cary, NC).

## Results

**Figure 1:**
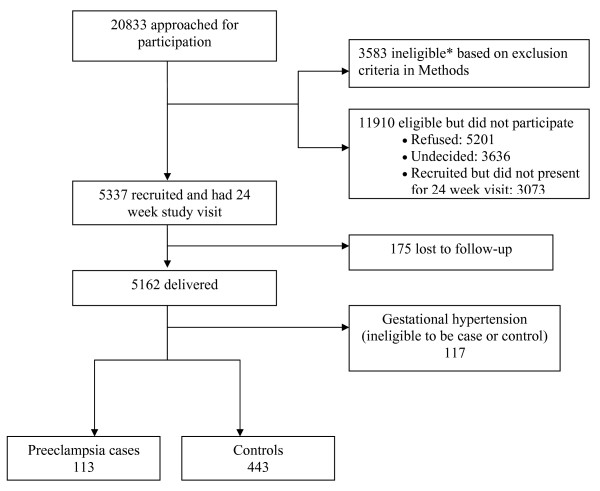
**Study flow diagram**. * data on reasons for ineligibility were available in 952. The most common reasons were lack of fluency in French or English (n = 321), plans to deliver in a non-study hospital (n = 272), severe chronic disease (n = 90) and gestational age at initial contact > 24 weeks (n = 62).

Of the 20,830 women who were approached for participation in the study from 1999 to 2003, 5337 were recruited and attended the 24-26-week study visit, and thus comprised the study cohort (Figure 1). Of these, 175 did not deliver at a study hospital and were lost to follow-up. Of 5162 study women followed to delivery, 113 developed preeclampsia (cases) (2.2%) (Table 1). Subsequent to identification of cases, 443 controls were chosen.

Characteristics of the study population are shown in Table 2. Controls had very similar characteristics to all non-cases (i.e. cohort women from among whom controls were selected). Women with preeclampsia had higher pre-pregnancy BMI than non-cases, were more likely to have a history of diabetes, prior hypertension and preeclampsia during a previous pregnancy and were less likely to be current smokers.

**Table 2:** Comparison of preeclampsia cases, non-cases and controls: demographic, medical and obstetric characteristics ascertained at 24-26 week study visit

Characteristic	Cases (n = 113)	Non-cases* (n = 5107)	Controls (n = 443)
Age category			
< 20 years	3%	3%	3%
20-34 years	78%	79%	79%
> 34 years	19%	18%	18%
Hospital recruited			
Hôpital St. Luc	15%	22%	24%
Hôpital Maisonneuve Rosemont	39%	36%	37%
Royal Victoria Hospital	22%	18%	17%
Jewish General Hospital	24%	24%	22%
Body Mass Index before pregnancy, kg/m^2^			
< 18.5	2%	8%	9%
18.5 to < 25	52%	63%	60%
25 to < 30	27%	18%	18%
≥ 30	19%	11%	13%
Primigravida	40%	35%	34%
Use of prenatal vitamins	92%	92%	92%
Diabetes before pregnancy or prior to 24 weeks gestation	6%	2%	1%
High blood pressure before pregnancy	8%	4%	3%
Previous preeclampsia or eclampsia (among women with prior pregnancy lasting > 20 wks)	21%	3%	3%
Smoke currently	10%	16%	16%

* Non-cases = study cohort (n = 5337) excluding cases (n = 113) and women who developed gestational hypertension without preeclampsia (n = 117).

Controls were selected from among non-cases as described under Methods.

Pregnancy outcomes are shown in Table 3. Cases with preeclampsia were significantly more likely than controls to deliver at earlier gestational age and to have a higher incidence of SGA birth, with correspondingly lower BWGA z-scores.

**Table 3:** Gestational age and fetal growth among preeclampsia cases and controls

	Cases	Controls	P value
Gestational age at delivery, weeks; mean (SD)*	36.8 (3.5)	39.2 (1.4)	< 0.0001
Gestational age at delivery, weeks*			
< 32 weeks	8 (7%)	1 (0%)	< 0.0001
32-33 weeks	6 (5%)	0 (0%)	
34-36 weeks	23 (21%)	18 (4%)	
≥ 37 weeks	76 (67%)	415 (96%)	
Birthweight, grams; mean (SD)^#^	2834.4 (848.4)	3485.3 (500.2)	< 0.0001
Z-score birthweight for gestational age^; mean (SD)	-0.31 (1.0)	0.15 (0.9)	< 0.0001
Small for gestational age^	22 (21.6%)	32 (7.3%)	< 0.0001
Large for gestational age^	6 (5.9%)	50 (11.3%)	0.10
Z-score placental weight for gestational age^+^; mean (SD)	-0.01(1.13)	0.15 (0.98)	0.25

Values shown are n (%) unless otherwise noted

* data missing for 9 subjects;^# ^data missing for 5 subjects;

^ Z-score birthweight for gestational age, small for gestational age, large for gestational age as defined in Kramer et al [14]; data missing for 13 subjects

^+ ^Z-score placental weight for gestational age calculated as defined by McNamara [15,16]; placenta data missing for 186 subjects

### Maternal smoking and preeclampsia

Self-reported smoking and hair nicotine levels in cases and controls are shown in Table 4. While results did not achieve statistical significance, cases tended to be less likely than controls to report smoking throughout pregnancy (p = 0.07) or to report smoking steadily within the same category of number of cigarettes throughout pregnancy (p = 0.05). Cases also had lower mean hair nicotine levels (p = 0.10) and reported smoking fewer cigarettes per day (p = 0.08) than controls.

**Table 4:** Self-reported cigarette smoking and hair nicotine levels in preeclampsia cases and controls

Variable	Cases N = 113	Controls N = 443	p-value
Smoked throughout pregnancy	10 (8.9%)	69 (15.6%)	0.07
Smoked steadily (within the same category of number of cigarettes) throughout pregnancy	4 (3.4%)	41 (9.3%)	0.05
Average number of cigarettes/day, mean (SD)*	1.20 (3.6)	1.91 (4.7)	0.08
Category of average number of cigarettes smoked per day			
0	90 (80.4%)	340 (77.3%)	0.69
1-10	16 (14.3%)	67 (15.2%)	
> 10	6 (5.4%)	33 (7.5%)	

Hair nicotine (ng/mg), mean (SD)^#^	1.29 (2.9)	1.94 (5.4)	0.10

Values shown are n (%) unless otherwise noted

* calculated as average of the number of cigarettes smoked per day in each of the trimesters.

^# ^Hair nicotine available for 99 cases and 398 controls

### Effect of smoking on fetal growth in controls

Among controls, mean (SD) BWGA z-score was lower in self-reported smokers than in non-smokers (-0.26 (0.89) vs. 0.22 (0.96); p = 0.0002) and was lower in women with hair nicotine levels above vs. below the median (0.01 (0.99) vs. 0.30 (0.95); p = 0.004). Similarly, the risk of SGA birth was significantly increased in self-reported smokers vs. non-smokers (OR 2.28 [95% CI 1.01, 5.56]) and in women with hair nicotine levels above vs. below the median (OR 5.36 [1.99, 14.40]).

### Association between smoking, preeclampsia and fetal growth

Consistent with the findings reported above, linear regression analysis showed that self-reported smoking and preeclampsia were each associated with lower BWGA z-scores (β = -0.29; p = 0.008, and β = -0.67; p < 0.0001, respectively). However, a significant positive interaction was detected between smoking and preeclampsia (interaction term β = +0.86; p = 0.0008) such that being a smoker decreased the BWGA z-score by -0.29 in controls but increased it by +0.57 in preeclampsia cases (Model 1a) (Table 5). In clinical terms, this increase in z-score would translate into a 234 g heavier weight for a male singleton born at 34 weeks, or a 255 g heavier weight for a male singleton born at 38 weeks [14]. Results were similar after adjustment for age, pre-pregnancy BMI, maternal language other than French or English, pre-gestational diabetes, chronic hypertension and parity (nullipara vs. other) (Model 1a adjusted), adjustment for PWGA (Model 1b), and adjustment for all of the above (Model 1b adjusted) (Table 5).

**Table 5:** Linear regression analyses: Relationship between maternal exposure to smoking, preeclampsia and fetal growth

Model	Unadjusted β (95% CI)	p-value	Adjusted β^#^ (95% CI)	p-value
**Model 1a** z-score BWGA = smoker + preeclampsia + interaction term				

Smoker	-0.29 (-0.51,-0.08)	0.008	-0.38 (-0.61, -0.15)	0.0013
Preeclampsia	-0.67 (-0.91,-0.43)	< 0.0001	-0.67 (-0.93, -0.41)	< 0.0001
Smoker* preeclampsia	0.86 (0.36, 1.36)	0.0008	0.86 (0.33, 1.39)	0.0015

**Model 1b** z-score BWGA = smoker + preeclampsia + interaction term + z-score PWGA				

Smoker	-0.30 (-0.50, -0.09)	0.004	-0.32 (-0.54, -0.09)	0.0067
Preeclampsia	-0.61 (-0.86, -0.35)	< 0.0001	-0.60 (-0.87, -0.33)	< 0.0001
Smoker* preeclampsia	0.74 (0.25, 1.23)	0.003	0.69 (0.16, 1.22)	0.0112
z-score PWGA	0.56 (0.48, 0.65)	< 0.0001	0.52 (0.43, 0.61)	< 0.0001

**Model 2a** z-score BWGA = log hair nicotine + preeclampsia + interaction term				

Log nicotine	-0.12 (-0.16, -0.05)	0.0004	-0.086 (-0.16, -0.01)	0.0234
Preeclampsia	-0.31 (-0.55, -0.07)	0.011	-0.36 (-0.63, -0.09)	0.0087
Log hair nicotine * preeclampsia	0.28 (0.11, 0.46)	0.002	0.26 (0.07, 0.46)	0.0091

**Model 2b** z-score BWGA = log hair nicotine + preeclampsia + interaction term + z-score PWGA				

Log nicotine	-0.09 (-0.16, -0.03)	0.005	-0.06 (-0.13, 0.01)	0.0912
Preeclampsia	-0.31 (-0.54, -0.08)	0.009	-0.33 (-0.60, -0.07)	0.0131
Log hair nicotine * preeclampsia	0.25 (0.07, 0.42)	0.007	0.25 (0.06, 0.44)	0.0095
z-score PWGA	0.56 (0.48, 0.64)	< 0.0001	0.52 (0.42, 0.62)	< 0.0001

Dependent variable for all models: z-score birthweight for gestational age (BWGA), as defined in Kramer et al [14]

PWGA = placental weight for gestational age; z-score PWGA calculated as defined by McNamara [15,16]

^# ^analyses adjusted for age, pre-pregnancy body mass index, maternal language other than French or English, diabetes, chronic hypertension and parity (nullipara vs. other). For adjusted models 1a and 1b, diabetes was statistically significant (p = 0.03 and p = 0.03, respectively). No other variables were statistically significant in any of the models.

R-square for models: Model 1a unadjusted, 0.057, Model 1a adjusted, 0.096; Model 1b unadjusted, 0.388, Model 1b adjusted, 0.391; Model 2a unadjusted, 0.062, Model 2a adjusted, 0.100; Model 2b unadjusted, 0.395, Model 2b adjusted, 0.384.

Similar results were obtained when log hair nicotine was substituted for self-reported smoking in the models (Models 2a and 2b) (Table 5). For example, in Model 2a, an increase in log hair nicotine by 1 unit decreased the BWGA z-score by -0.12 in controls, but increased the BWGA z-score by +0.16 in preeclampsia cases (i.e. βnic (log nicotine) + βinteraction (log nicotine*preeclampsia) = -0.12*1 + 0.28 (1*0) = -0.12 for controls, and -0.12*1 + 0.28 (1*1) = 0.16 for preeclampsia cases).

Due to the small numbers of smokers in subgroups of cases with early onset preeclampsia and severe preeclampsia, we were unable to examine the association between smoking, subtypes of preeclampsia and fetal growth.

### Levels of sFlt-1 in association with preeclampsia and smoking

Mean (SD) plasma sFlt-1 was 761.5 (445.6) pg/ml in preeclampsia cases and 703.83 (358.8) pg/ml in controls (p = 0.21), and cases were more likely to have sFlt-1 concentrations in the top quartile of values than controls (33.9% vs. 21.9%, p = 0.016). Among preeclampsia cases, women with hair nicotine levels above the median had significantly lower mean (SD) sFlt-1 levels than women with hair nicotine levels below the median (660.4 (377.2) pg/ml vs. 903.5 (466.4) pg/ml, respectively; p = 0.0054) and tended to be less likely to have sFlt-1 concentrations in the top quartile of control values (29.4% vs. 44.7%, p = 0.16). Among controls, sFlt-1 levels did not vary by hair nicotine level.

## Discussion

We found that cigarette smoking was associated with reduced fetal growth restriction in preeclamptic women. This finding remained robust after adjustment for potentially confounding covariates. We further found that preeclamptic women who had higher levels of exposure to smoking, indicated by higher hair nicotine levels, had significantly lower plasma concentrations of the circulating anti-angiogenic protein sFlt-1 at 24-26 weeks gestation than preeclamptic women with lower levels of smoking exposure.

We also confirmed previous reports that smokers are less likely to develop preeclampsia than non-smokers and have lower birthweight for gestational age babies; that high BMI, diabetes, hypertension, primigravidity, previous preeclampsia and family history of preeclampsia increase the risk of preeclampsia; and that fetal outcomes are worse in preeclamptic vs. non-preeclamptic women.

Our study has a number of strengths. Cohort women were recruited prospectively and consecutively at four large maternity hospitals in Montreal that serve a wide socioeconomic and demographic spectrum and are likely to be broadly representative of the general obstetric population. While the incidence of preeclampsia in our cohort (2.2%) was at the lower end of that previously reported in healthy pregnant women [18], this likely reflects our use of strict, high-specificity criteria to diagnose preeclampsia. We are confident that we did not miss cases of preeclampsia as trained research assistants reviewed the delivery of each cohort woman to ascertain, using a detailed checklist, if criteria for preeclampsia were met. Our success in obtaining "true" cases of preeclampsia is shown by the differences in frequency of recognized preeclampsia risk factors and indicators of poorer pregnancy outcomes in cases and controls. Results were similar whether we assessed smoking exposure by self-report or using hair nicotine level, hence misclassification of smoking exposure is unlikely [19]. We used hair nicotine rather than hair cotinine as a measure of maternal tobacco smoke exposure because previous work by our group found that hair nicotine was a better predictor of reduced infant birthweight in control women, and hence a better measure of maternal smoke exposure [20]. The proportion of women who reported smoking during pregnancy was similar to that of other large cohorts of pregnant women [21,22]. We assessed fetal growth on a continuous scale adjusted for sex and gestational age, using Canadian population-based standards [14].

As the sample size of our study was predetermined by the requirements of the Montreal Prematurity Study, precision was limited for estimates of association between smoking and preeclampsia. Furthermore, we could not perform analyses by preeclampsia subtype, which would have been of interest as severe, early-onset preeclampsia may be a different disease entity from milder forms of preeclampsia [1]. We did not have information on race/ethnicity of study women, hence could not examine potential effects on BWGA z-score, preeclampsia incidence or hair nicotine levels. Finally, we acknowledge that associations should be considered hypothesis-generating and do not prove causality.

Smoking has consistently been shown to reduce the risk of preeclampsia by ~30% [2,3]. The biological mechanism of this protective effect is uncertain, but may relate to inhibitory effects of smoking by-products such as nicotine and carbon monoxide on endothelial dysfunction and excessive maternal inflammatory response [6]. Nicotine acts on human placenta to release placental acetylcholine, which stimulates release of endothelium-derived relaxing factor and nitric oxide [23], and in vitro studies show that nicotine selectively inhibits thromboxane A2 synthesis [24]. Both effects would be expected to reduce endothelial dysfunction and preserve placental blood flow. Nicotine also has anti-inflammatory activity and inhibits placental cytokine production [4]. Carbon monoxide has direct placental effects that could reduce the risk of preeclampsia, including promotion of trophoblast invasion, reduced decidual inflammatory response, increased uteroplacental blood flow, decreased hypoxia-induced apoptosis and upregulation of placental antioxidant systems [5,25]. Finally, findings of a recent Swedish birth register study suggest that tobacco combustion products rather than nicotine may be protective of preeclampsia, and it may be the smoking habits in middle or late gestation (rather than beginning of pregnancy) that influence the risk of developing preeclampsia [26].

Our finding that smoking attenuated the relationship between preeclampsia and fetal growth restriction is intriguing, as some studies have reported a U-shaped pattern of fetal growth in populations of women with preeclampsia. In a population-based, retrospective Canadian study, Xiong reported that women with preeclampsia had a 2.6-fold higher risk of SGA birth than normotensive women, but also had a 1.8-fold higher risk of LGA birth [9]. While analyses controlled for self-reported smoking, potential interaction between smoking and preeclampsia on fetal growth was not examined. Similar results were reported in a population of Chinese women, for which information on maternal smoking was not available [27]. Based on our finding that preeclamptic women who smoked had infants with higher BWGA compared to preeclamptic women who were non-smokers, we suggest that smoking may account, at least in part, for the U-shaped pattern of fetal growth in preeclampsia.

In our study, plasma sFlt-1 levels were significantly lower in preeclamptic women with higher hair nicotine levels. This suggests that smoking may attenuate fetal growth restriction by reducing production of the anti-angiogenic protein sFlt-1. sFlt-1 is secreted by the placenta into the maternal circulation and adheres to the receptor-binding domains of placental growth factor and vascular endothelial growth factor (VEGF), preventing interaction with endothelial receptors, blocking VEGF-mediated vasodilation and inducing endothelial dysfunction [8], considered to be key to the pathogenesis of preeclampsia [6]. Levels of sFlt-1 are elevated in preeclamptic women, and beginning at 21-24 weeks gestation, in pregnant women who subsequently develop preeclampsia [7,8]. Smoking increases placental expression of VEGF [28], and plasma sFlt-1 levels have been found to be decreased in smokers [29]. Furthermore, carbon monoxide has inhibits sFlt-1 production in mice [30]. This effect of smoking on increasing pro-angiogenic factors and reducing anti-angiogenic factors may explain why preeclampsia occurs less frequently in smokers, and could also explain our finding that among preeclamptic women, smoking limits fetal growth restriction. A recent study of 58 preeclamptic women also reported a tendency to lower sFlt-1 levels in smokers vs. non-smokers [7].

Finally, our finding of a protective effect of smoking on preeclampsia-associated fetal growth restriction corroborates that of a recent linked record study of > 650,000 pregnancies, in which self-reported smokers who developed preeclampsia had a lower adjusted mean difference in birthweight compared to controls than would have been expected based on the independent additive effects of smoking and preeclampsia, and lower than expected rates of preterm delivery [31]. Furthermore, a secondary analysis of the Calcium for Preeclampsia Prevention trial reported that among 274 nulliparous women with preeclampsia, smoking did not act to further reduce infant birthweight, compared with non-smoking women [32].

## Conclusions

In conclusion, fetal growth restriction in preeclamptic pregnancies seems to be attenuated by maternal smoking. Smoking may account, at least in part, for the U-shaped pattern of fetal growth described in women with preeclampsia, and may exert this effect by reducing levels of the anti-angiogenic protein sFlt-1. Further exploration of the reasons why smoking may protect against preeclampsia-associated fetal growth restriction could increase understanding of the pathophysiology of preeclampsia.

## Competing interests

The authors declare that they have no competing interests.

## Authors' contributions

SRK designed the study and directed its implementation, including acquisition of data and quality assurance, and drafted and revised the article. NDA contributed to the conception of the study, performed some of the analyses and reviewed and helped to revise the manuscript. HMN contributed to the conception of the study, and reviewed and helped to revise the manuscript. GK performed the hair nicotine analyses and reviewed and helped to revise the manuscript. JG Jr performed the biomarker analyses and reviewed and helped to revise the manuscript. MD performed the statistical analyses and reviewed and helped to revise the manuscript. RWP helped to design the study's analytic strategy and reviewed and helped to revise the manuscript. MSK contributed to the conception of the study and the acquisition of data, provided administrative support, helped to design the study's analytic strategy and reviewed and helped to revise the manuscript.

All authors read and approved the final manuscript.

## Pre-publication history

The pre-publication history for this paper can be accessed here:

http://www.biomedcentral.com/1471-2393/11/91/prepub
